# Creating a Mentoring Programme: Kent and Medway Psychiatry Undergraduate Scheme (KAMPUS)

**DOI:** 10.1192/bjo.2025.10325

**Published:** 2025-06-20

**Authors:** Maham Zahid, Sharna Bennett, Tiago Gameiro-Inacio, Joanne Rodda

**Affiliations:** 1 Kent and Medway Partnership Trust (KMPT), Maidstone, United Kingdom; 2 KMPT, Canterbury, United Kingdom; 3 KMPT, Dartford, United Kingdom; 4 Kent and Medway Medical School, Canterbury, United Kingdom

## Abstract

**Aims:** The Kent and Medway Psychiatry Undergraduate Scheme (KAMPUS) aims to provide medical students with experience in psychiatry at an early stage in their training, and psychiatry trainees the opportunity to develop mentoring and leadership skills.

Early exposure to psychiatry improves student perceptions of the specialty. KAMPUS is based on a similar programme in a London medical school which has reported positive outcomes. KAMPUS was adapted for the structure and geography of the local medical school and mental health trust.

**Methods:** A committee of psychiatrists, trainees and student representatives co-developed KAMPUS and wrote handbooks for students, trainees and clinical supervisors. Year 1–2 students and core psychiatry trainees were invited to participate. 18 students and 15 trainees registered for KAMPUS in the 2023–2024 academic year. Lead mentors supported small groups of students virtually, and clinic-based mentors were trainees based near the school, who provided clinical shadowing opportunities. Combined educational/social events were organised in collaboration with the Psychiatry Society, and a formal day of mentoring training was provided for trainees.

**Results:** Trainees provided regular mentoring and shadowing opportunities. Lead mentor group discussion topics included training pathways, case presentations and practice exam questions. Two educational/social events were attended by students and trainees. Trainees gave positive feedback regarding mentoring training.

**Conclusion:** KAMPUS is deliverable across the wide geographical area covered by the medical school and mental health trust in Kent. It has provided early experience in psychiatry for students and a development opportunity for trainees, with positive initial feedback.

